# Deletion of BmoR affects the expression of genes related to thiol/disulfide balance in *Bacteroides fragilis*

**DOI:** 10.1038/s41598-018-32880-7

**Published:** 2018-09-26

**Authors:** Felipe L. Teixeira, Heidi Pauer, Scarlathe B. Costa, C. Jeffrey Smith, Regina M. C. P. Domingues, Edson R. Rocha, Leandro A. Lobo

**Affiliations:** 1 Departamento de Microbiologia Médica, Instituto de Microbiologia Paulo de Góes, Universidade Federal do Rio de Janeiro, Rio de Janeiro, RJ Brazil; 2 Department of Microbiology and Immunology, Brody School of Medicine, East Carolina University, Greenville, NC USA

## Abstract

*Bacteroides fragilis*, an opportunistic pathogen and commensal bacterium in the gut, is one the most aerotolerant species among strict anaerobes. However, the mechanisms that control gene regulation in response to oxidative stress are not completely understood. In this study, we show that the MarR type regulator, BmoR, regulates the expression of genes involved in the homeostasis of intracellular redox state. Transcriptome analysis showed that absence of BmoR leads to altered expression in total of 167 genes. Sixteen of these genes had a 2-fold or greater change in their expression. Most of these genes are related to LPS biosynthesis and carbohydrates metabolism, but there was a significant increase in the expression of genes related to the redox balance inside the cell. A pyridine nucleotide-disulfide oxidoreductase located directly upstream of *bmoR* was shown to be repressed by direct binding of BmoR to the promoter region. The expression of two other genes, coding for a thiosulphate:quinone-oxidoreductase and a thioredoxin, are indirectly affected by *bmoR* mutation during oxygen exposure. Phenotypic assays showed that BmoR is important to maintain the thiol/disulfide balance in the cell, confirming its relevance to *B*. *fragilis* response to oxidative stress.

## Introduction

*Bacteroides fragilis* is a strict anaerobe found in the gut microbiota of humans. It is the opportunistic pathogen most frequently isolated from extra-intestinal anaerobic infections^1^, including highly oxygenated tissues such as the peritoneal cavity. Although anaerobic bacteria typically cannot grow in oxygen concentrations higher than 5 µM dissolved O_2_^2^, *Bacteroides* spp. have developed mechanisms to induce an oxidative stress response (OSR) necessary to maintain viability under oxidative stress conditions^3,4^. The expression of an extensive number of proteins associated with the OSR mechanism confers high aerotolerance to *B*. *fragilis* and enables survival for several days during exposure to atmospheric oxygen^5–7^.

*B*. *fragilis* OSR repertoire includes enzymes such as catalase (KatB)^5^, peroxidases (AhpCF, Tpx, Ccp1)^8,9^, superoxide dismutase (Sod)^10^ and iron storage proteins (Dps, DpsL, FtnA)^11,12^. *B*. *fragilis* also possesses genes coding for 6 different thioredoxin (Trx) which are small redox proteins associated with thiol-disulfide balance in the cell^13^. Moreover, the presence of a cytochrome *bd* oxidase (CydAB) allows for growth on nanomolar (500 nM dissolved O_2_ or 0.05% oxygen) concentrations of O_2_^2^. This robust OSR is essential for *B*. *fragilis* proliferation in oxygenated tissues such as the peritoneal cavity during the initial steps of abscess formation^2^ by contributing to resistance against the oxidative burst of neutrophils and macrophages from the host immune defenses.

During oxidative stress conditions, the expression of 45% of the coding sequences in *B*. *fragilis* genome are altered, which indicates a major remodeling of cellular physiology. Stressed cells show reduced expression in genes related to translation, cell wall biogenesis, nucleotide metabolism and coenzyme metabolism. On the other hand, oxidative stress induces energy production and carbohydrate metabolism pathways, with upregulation of genes encoding enzymes from oxidative pathways, such as citric acid cycle, pentose phosphate pathway and glycolysis, which could contribute to regeneration of reducing agents (such as NDAPH/NADH) that are depleted during the cell response to oxidative conditions. The expression of at least 30 genes directly related to *B*. *fragilis* OSR (detoxification, DNA/protein repair, redox balance) is affected by oxidative stress^3^. This data indicates that a strong regulatory network is needed to coordinate such complex response. So far, few regulators associated with the OSR have been characterized. The LysR family regulator of the peroxide response, OxyR, has been identified as one of these regulators which controls the expression of five genes related to hydrogen peroxide detoxification (*katB*, *ahpC*, *ahpF*, *tpx* and *dps*)^3,8,9,14,15^.

More recently, a member of the MarR family of transcriptional regulators named BmoR has also been described to participate in the regulation of the OSR in *B*. *fragilis*. However, the genes regulated by BmoR have not been identified^16^. MarR, the prototypical member of this protein family, was described as the regulator of a multiple antibiotic resistance (*mar*) locus in *Escherichia coli* in 1983^17,18^. Since then, several regulators with similar structure have been added to the family^19^. Regulation by members of the MarR family comprises a wide range of physiological processes in both archaea and bacteria, including expression of virulence genes, antimicrobial resistance and resistance to oxidative stress^20,21^. In *B*. *fragilis*, besides BmoR, at least three other members of MarR have been annotated^22^. Two of them, MarRI and MarRII, have been associated with both oxidative stress and antimicrobial resistance^23^.

In this study, we investigate the role of BmoR in the OSR of *B*. *fragilis*. We used microarray gene expression to demonstrate the effect of *bmoR* deletion on gene expression profile of *B*. *fragilis* grown under anaerobic conditions compared to oxidative stress. The phenotype of the isogenic *bmoR* mutant was also evaluated. Our results show that BmoR regulates BF638R_0572 and the *trxP* operon, a set of genes involved in the maintenance of intracellular redox state, particularly the thiol/disulfide balance of the cell.

## Results

### Deletion of bmoR severely impacts the expression of genes related to redox balance

In a previous study, we established a correlation between BmoR and the OSR of *B*. *fragilis*^16^. To understand the impact of this regulator during the OSR, we compared the transcriptome response of wild-type and *ΔbmoR* mutant strains growing in anaerobiosis and after 1 h of oxygen exposure. Deletion of *bmoR* affected the expression (p < 0.05) of 341 genes: 167 genes in the anaerobic condition (Tables S3 and S4) and 187 genes during oxygen exposure (Tables S5 and S6), with an overlap of only 13 genes with altered expression on both conditions (Tables S7). Among the 167 genes affected in anaerobiosis, 16 had at least a 2-fold change in the expression; eight were upregulated and eight were downregulated (Table 1). The most expressive change (11-fold upregulation in the mutant strain) was in the gene BF638R_0572, located directly upstream of *bmoR*. Besides BF638R_0572, most of the genes altered in this condition were related to LPS biosynthesis and carbohydrates metabolism. The most expressive changes (>5-fold upregulation in the mutant strain) found among the 187 genes altered on the *ΔbmoR* mutant after oxygen exposure were in the genes BF638R_0572, BF638R_2699, BF638R_2700, BF638R_2701 and BF638R_4194. Four other genes presented at least a 2-fold change, two were upregulated and two were downregulated (Table 2), these genes are related to either LPS biosynthesis or carbohydrates metabolism.

**Table 1:** Genes up- or down regulated at least 2-fold in *B. fragilis bmoR* mutant strain in anaerobiosis.

Gene ID	GenBank definition	Fold-change
***Upregulated***		
BF638R_0572	putative pyridine nucleotide oxidoreductase	11.357
BF638R_1879	putative LPS biosynthesis related epimerase	3.467
BF638R_1878	putative LPS biosynthesis related dehydratase	3.186
BF638R_1868	putative LPS biosynthesis related phosphoenolpyruvate decarboxylase	2.77
BF638R_1864	putative glucose-1-phosphate thymidyl transferase	2.767
BF638R_1873	putative LPS biosynthesis related hypothetical protein	2.731
BF638R_1871	putative LPS biosynthesis related acetyltransferase	2.724
BF638R_1866	putative glucose-1-P-cytidylyltransferase	2.465
***Downregulated***		
BF638R_1439	putative transmembrane protein	2.43
BF638R_2599	putative transcriptional regulatory protein	2.292
BF638R_1435	putative UDP-GlcNAc 2-epimerase	2.115
BF638R_1454	putative LPS biosynthesis related glucose-1-phosphate thymidylyltransferase	2.103
BF638R_2735	conserved hypothetical protein	2.077
BF638R_3479	putative LPS biosynthesis related glycosyltransferase	2.046
BF638R_1440	putative transmembrane protein	2.022
BF638R_1441	hypothetical protein	2.022

**Table 2:** Genes up- or down regulated at least 2-fold in *B*. *fragilis bmoR* mutant strain after 1 hour of exposure to atmospheric oxygen.

Gene ID	GenBank definition	Fold-change
***Upregulated***		
BF638R_2699	hypothetical membrane protein	7.868
BF638R_0572	putative pyridine nucleotide oxidoreductase	7.623
BF638R_2700	conserved hypothetical membrane protein	7.411
BF638R_4194	putative lipoprotein	6.686
BF638R_2701	putative exported thioredoxin	5.104
BF638R_4193	putative outer membrane protein	2.729
BF638R_1869	putative LPS biosynthesis related 2-aminoethylphosphonate pyruvate aminotransferase	2.509
***Downregulated***		
BF638R_0786	putative LPS biosynthesis related glycosyl transferase	2.472
BF638R_2591	putative polysaccharide transporter/flippase	2.038

Our transcriptomic analysis revealed that deletion of *bmoR* had no impact on the expression of major detoxifying enzymes, such as catalase, alkyl hydroperoxide reductase and superoxide dismutase. Nevertheless, among the genes with altered expression after oxygen exposure, at least three of them are related to the redox balance in the cell: BF638R_0572 and BF638R_2700 code oxidoreductases and BF638R_2701 (*trxP*) codes a thioredoxin. According to the literature, *trxP* is the only gene associated to *B*. *fragilis* OSR, but no clear role for him has been presented yet^3,13,24^. Therefore, we decided to analyze all of those genes further, both *in silico* and *in vitro*, to understand their role in the cell and their relation to *bmoR*.

We used the amino acid sequence of the proteins coded by those genes to identify conserved domains based on Pfam and TIGRFAM databases (Table 3). BF638R_0572 codes for a pyridine nucleotide-disulfide oxidoreductase with four conserved domains: a coenzyme A (CoA)-disulfide reductase domain, a rhodanese-like domain, a sulfurtransferase TusA domain and a DsrE/DsrF/DrsH-like family domain (Fig. S1). BF638R_2699, the first gene of the operon BF638R_2699-2701, codes for a hypothetical protein with no conserved domains or predicted function. The membrane protein coded by BF638R_2700 (DoxDA) is a thiosulfate:quinone oxidoreductase (TQO) with two subunits, the conserved domains DoxD and DoxA. BF638R_2701 (TrxP) codes for an exported thioredoxin (Fig. S1).

**Table 3:** Conserved domains of potential OSR-related proteins based on Pfam and TIGRFAM databases.

Conserved domains	Accession	Interval	E-value
***BF638R_0572***			
CoA-disulfide reductase	TIGR03385	14–440	2.02e-153
Rhodanese-like	pfam00581	462–540	4.28e-19
Sulfurtransferase TusA	pfam01206	589–657	7.83e-21
DsrE/DsrF/DrsH-like family	pfam13686	671–825	4.58e-60
***BF638R_2699***			
—	—	—	—
***BF638R_2700***			
TQO small subunit DoxD	pfam04173	19–185	2.52e-66
TQO small subunit DoxA	pfam07680	211–341	1.63e-69
***BF638R_2701***			
Thioredoxin	pfam00085	43–156	1.54e-33

**Figure 1:**
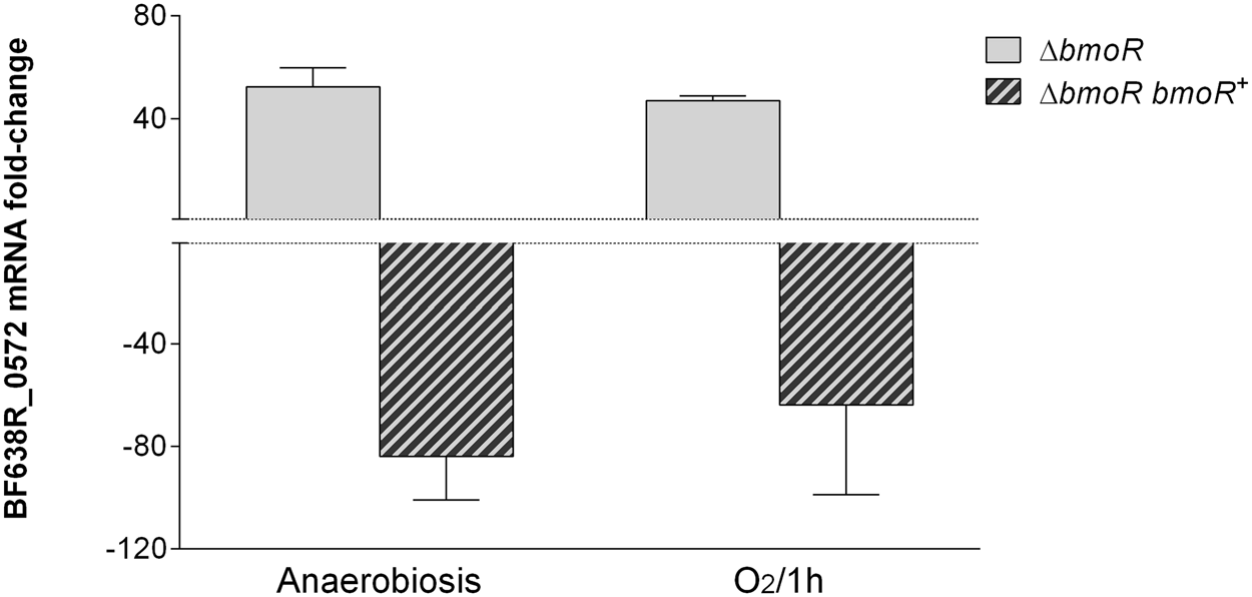
RT-qPCR expression analysis of the pyridine nucleotide-disulfide oxidoreductase coded by BF638R_0572 in *B*. *fragilis* 638R mutant strains grown in anaerobiosis and after exposure to atmospheric oxygen for 1 hour. RNA was isolated from mutant and parental strains for each condition and it was followed by RT-qPCR analysis. Results are expressed as fold change relative to expression levels in the parental strain under each condition.

**Figure 2:**
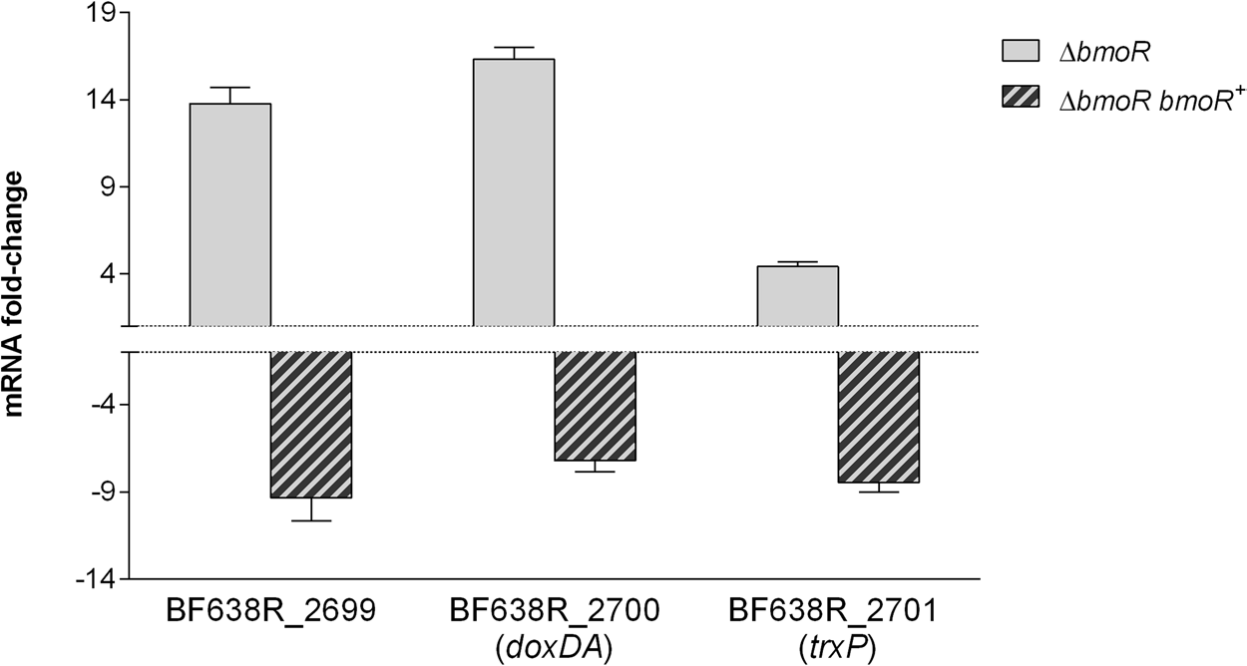
RT-qPCR expression analysis of the operon BF638R_2699-2701 in *B*. *fragilis* 638R mutant strains exposed to atmospheric oxygen for 1 hour. RNA was isolated from mutant and parental strains and it was followed by RT-qPCR analysis. Results are expressed as fold change relative to expression levels in the parental strain.

**Figure 3:**
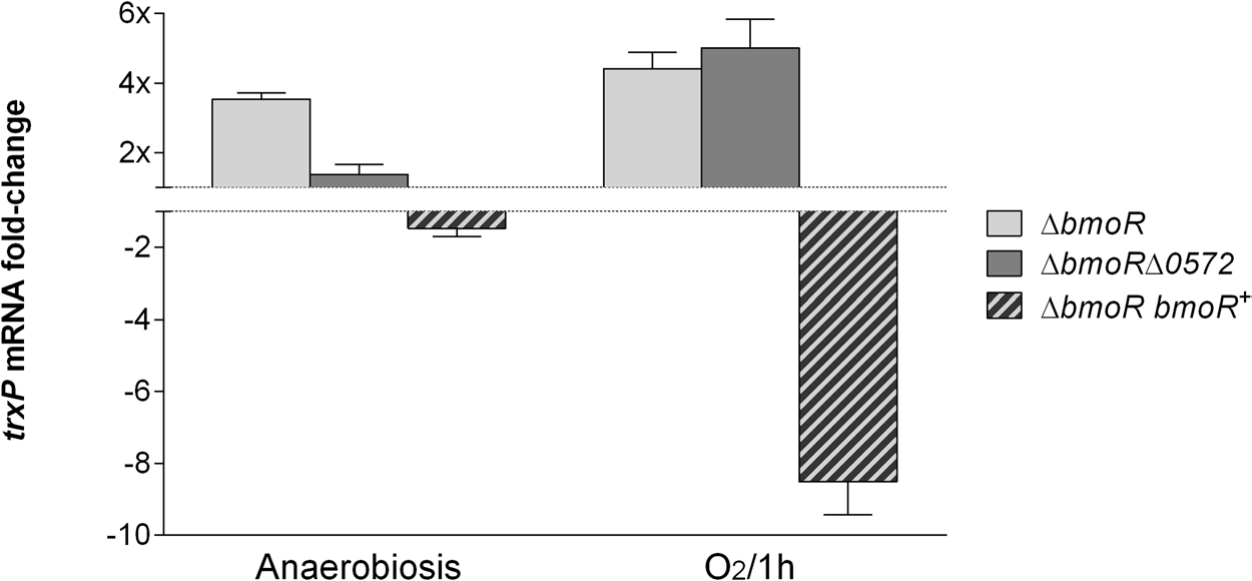
RT-qPCR expression analysis of *trxP* in *B*. *fragilis* 638R mutant strains grown in anaerobiosis and after exposure to atmospheric oxygen for 1 hour. RNA was isolated from mutant and parental strains for each condition and it was followed by RT-qPCR analysis. Results are expressed as fold change relative to expression levels in the parental strain under each condition.

The expression of those genes was analyzed by RT-qPCR during anaerobiosis or after exposure to oxygen. The gene BF638R_0572 was upregulated 50-fold in both aerobic and anaerobic conditions on the Δ*bmoR* mutant compared to the wild-type strain (Fig. 1). The genes BF638R_2699, *doxDA* and *trxP* were all upregulated on the Δ*bmoR* mutant during oxygen exposure (approximately 14-, 16 and 4-fold, respectively) compared to wild-type (Fig. 2). Evaluation of *trxP* individually (Fig. 3) showed that *bmoR* deletion could also affect its expression during anaerobiosis and that double mutation of *bmoR* and BF638R_0572 affects *trxP* expression during oxygen exposure, but not under anaerobiosis.

Complementation of BmoR led to reversion of the expression pattern observed instead of recovering the expression of the genes to wild-type level. BF638R_0572 was downregulated 60- and 80-fold during oxygen exposure and anaerobiosis, respectively (Fig. 1); and the operon BF638R_2699-2701 was downregulated around 9-, 7- and 8.5-fold, respectively (Fig. 2). This might have happened because standard plasmid complementation does not always correct the mutation due to presence of multiple copies of the complemented gene^3^.

### BmoR binds to its own promoter region to repress the expression of its operon

**Figure 4:**
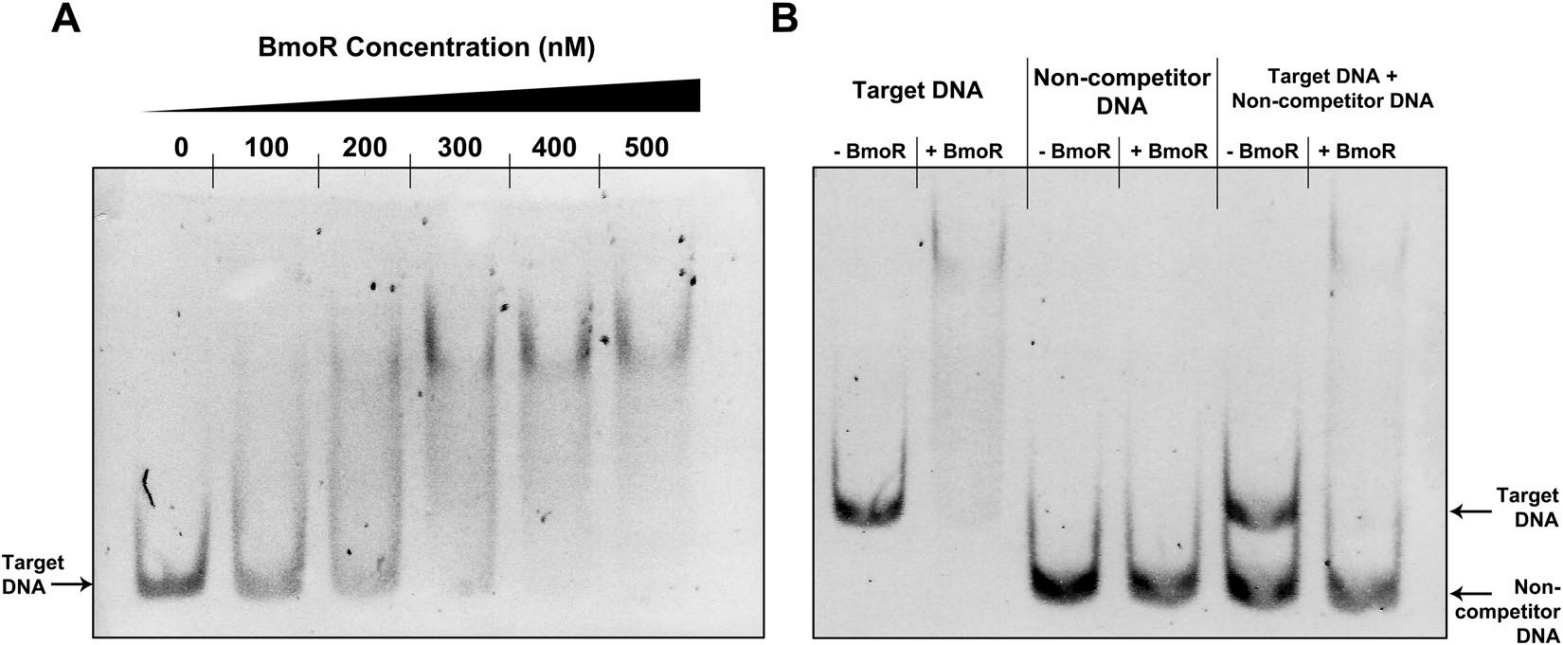
Electrophoretic mobility shift assay showing binding of BmoR to its own promoter region. (**A**) Higher concentrations of BmoR leads to increased binding to the target DNA. (**B**) Binding of BmoR to target DNA is specific and not affected by a nonspecific DNA sequence.

Upregulation of several genes in the absence of *bmoR* indicates that BmoR might be involved in negative regulation of these genes. Electrophoretic mobility shift assay (EMSA) was performed to investigate binding of BmoR to the promoter region of potential targets, such as BF638R_0572, BF638R_2699 and BF638R_4194 promoter regions. A 151 bp fragment containing BF638R_0572-*bmoR* operon promoter was used as target-DNA to confirm that BmoR binds its own operon promoter, indicating that *bmoR* expression is self-regulated. A protein-DNA complex was observed after interaction with 400 nM of purified BmoR and target DNA as determined by EMSA (Fig. 4A). BmoR does not bind to a nonspecific DNA sequence used as non-competitor DNA, indicating that the *bmoR* promoter region contain specific nucleotide sequence recognized by BmoR (Fig. 4B). No protein-DNA band shift was observed on the EMSA performed with BF638R_2699-2701 operon and BF638R_4194 promoter regions (data not shown). This suggests that altered expression of BF638R_2699-2701 and BF638R_4194 genes in the *bmoR* mutant background might be due to an indirect regulatory effect.

### Phenotypic analysis reveals a role for BmoR operon on thiol/disulfide balance, with no significant impact on B. fragilis survival during oxygen exposure

**Figure 5:**
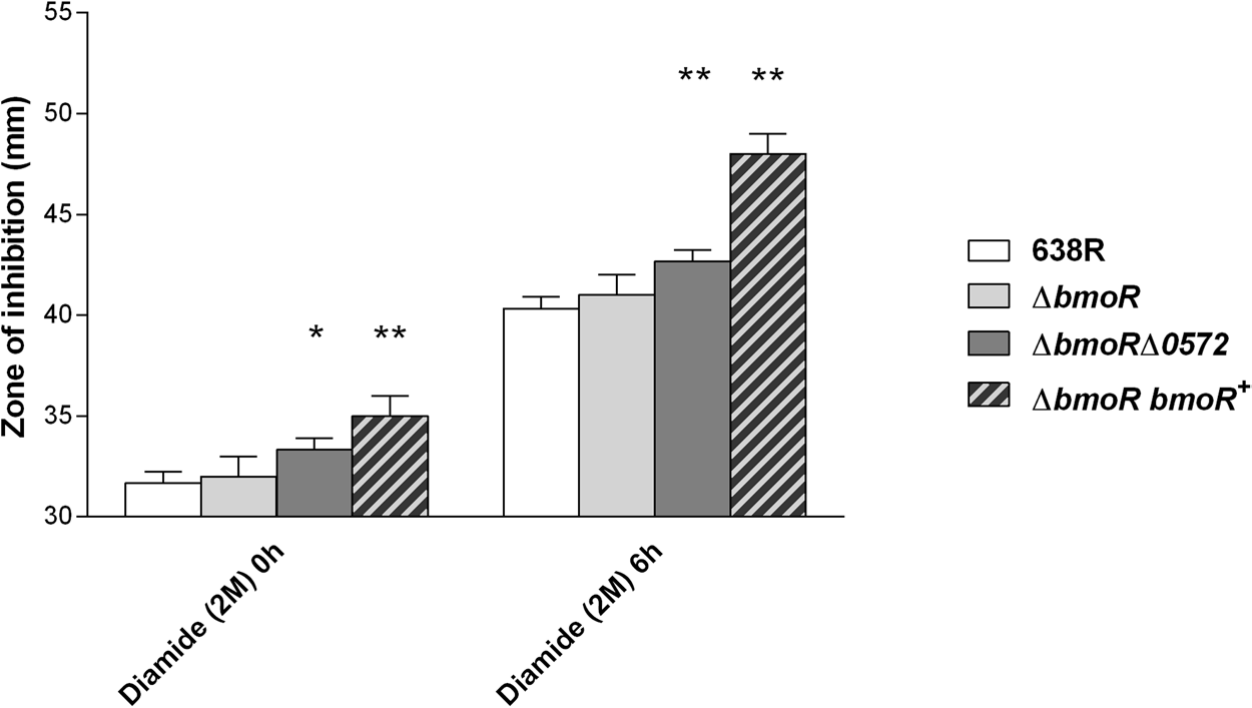
Disk diffusion assay with the thiol oxidant diamide. The diameter of the inhibition halo formed by diamide impregnated disks was measured after incubation in anaerobic chamber at 37 °C for 48 h (or 42 h after a 6 h aerobic incubation for half of the cultures). Significative difference (*p < 0.05; **p < 0.005) between mutant and wild-type strains was determined by t test.

Resistance to diamide, a thiol oxidant, was tested by disk diffusion assays. Although the absence of BmoR did not affect resistance against diamide when comparing Δ*bmoR* mutant to wild-type strain (p > 0.05) (Fig. 5), the double mutant Δ*bmoR*Δ*0572* was significantly impaired in both zero (p = 0.007) and six (p = 0.001) hours of incubation, implying a role of the operon on thiol/disulfide balance. On the other hand, complementation of Δ*bmoR* had a severe impact on resistance to diamide (Fig. 5) after zero (p = 0.001) and six (p < 0.001) hours of incubation.

**Figure 6:**
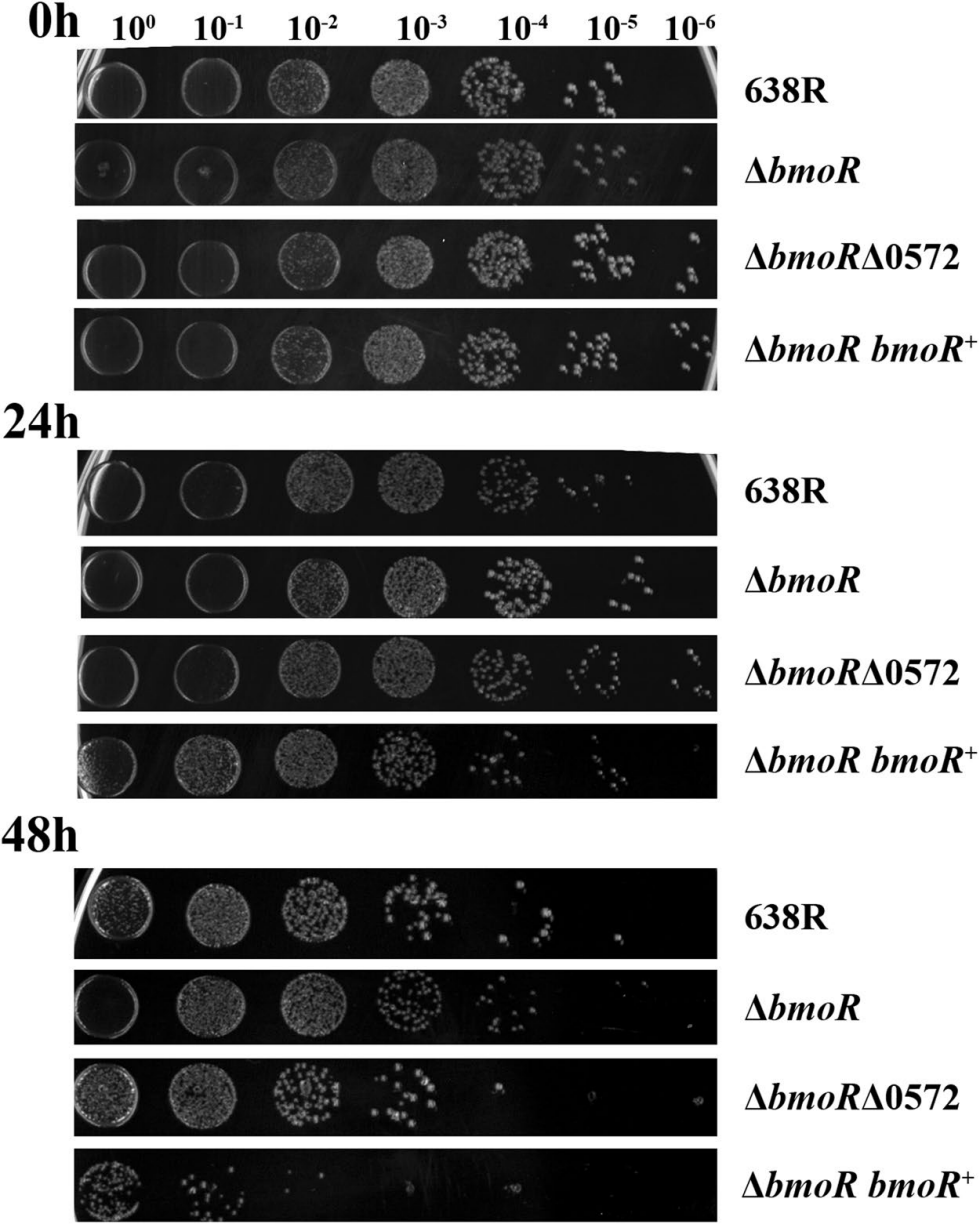
Survival of *B*. *fragilis* during extended exposure to atmospheric oxygen. Serial dilutions of wild-type and mutant strains culture were inoculated in BHI agar and exposed to oxygen for 24, 48 or 72 h, followed by anaerobic incubation. No growth was seen at 72 h of exposure.

Cell survival during prolonged exposure to atmospheric oxygen was also evaluated. None of the strains could survive for 72 h in an aerobic environment (data not shown). Survival of *B*. *fragilis* was not affected by *bmoR* or BF638R_0572 deletion. Growth of Δ*bmoR* and Δ*bmoR*Δ0572 strains showed no difference when compared to wild-type strain (Fig. 6). On the other hand, complementation of *bmoR* in the mutant strain caused a decrease in survival, with a difference of two orders of magnitude between Δ*bmoR bmoR*+ and wild-type strain after 48 h of oxygen exposure (Fig. 6).

## Discussion

The versatility of *B*. *fragilis* as both a commensal member of the gut microbiota or opportunist pathogen in endogenous infections provides an interesting model of study. To achieve this transition, this species must overcome several barriers, including oxidative stress. Although several OSR mechanisms have been described in the last two decades^2,3,5,9,11,13,15,16,25^, a complete picture of the regulation of such robust response is still missing. Thus, our aim was to advance the understanding of the species OSR, focusing on the importance of BmoR to the cell during the stress response.

The genetic complexity of *B*. *fragilis* OSR was first demonstrated by transcriptomic analysis that showed that almost half of its genes are amenable to changes in expression upon exposure to different oxidative agents^3^. We replicated the microarray analysis during oxygen exposure using a *B*. *fragilis* strain with deleted *bmoR*. A strong contrast was seen between our analysis on a *bmoR* mutant and the previously described transcriptomics of *oxyR* mutants. First, there is no overlap between the genes most affected by the individual mutations. Unlike OxyR, that already has an established regulon and inducing conditions^3^, no information was available for BmoR. Second, while knockout of the transcriptional activator OxyR causes an abrupt drop on the expression of regulated genes during exposure to oxygen^3^, deletion of *bmoR* causes strong upregulation of its putative target genes. This was the first clue suggesting that BmoR acts as a transcriptional repressor in *B*. *fragilis*.

The prototypical multiple antibiotic regulator MarR was described in *E*. *coli* as regulator of the operon *marRAB*. MarR binds to inverted repeats in specific sites inside the operator *marO*. In *E*. *coli*, each MarR subunit recognizes a TTGCC pentanucleotide in each inverted repeat, binding to the DNA with a dimeric conformation^17,26,27^. Analysis of the region upstream from *bmoR* revealed inverted repeats containing the pentanucleotide TTTCC in the promoter region of BF638R_0572 (Fig. S2). Though *marO* of *E*. *coli* contains two binding sites for the MarR dimer^27^, alignment between *marO* and the operator region of BmoR showed that only one site with inverted repeats is available for binding of BmoR (Fig. S2). Specific binding to the promoter region of BF638R_0572 was confirmed by EMSA and expression analysis showed that the absence of BmoR leads to upregulation of the operon. We concluded that BmoR binds to its own promoter region to repress itself (like MarR in *E*. *coli*^20^) and BF638R_0572.

The role of BmoR on the expression of the operon BF638R_2699-2701 remains elusive. EMSA analysis could not confirm binding of BmoR to the operon promoter region and no inverted repeats were detected on its sequence (data not shown), but RT-qPCR confirmed upregulation of BF638R_2699-2701 on the *bmoR* mutant strain, suggesting that BmoR regulation of this operon might occur indirectly. Expression of *trxP* on the double mutant revealed that upregulation in the absence of oxygen is reverted when mutation of BF638R_0572 is added to *bmoR* deletion. The oxidoreductase coded by BF638R_0572 contains a rhodanese domain and oxidation of thioredoxins by rhodaneses has been previously described in the literature^28,29^. Concomitant upregulation of BF638R_0572 and *trxP* suggests that the proteins coded by those genes may act in related pathways or even interact with each other. Additionally, a previous work with *Metallosphaera cuprina* TusA and DrsE, domains present in BF638R_0572, showed that those proteins could mobilize thiosulfate from tetrathionate^30^. According to KEGG pathway for sulfur metabolism in *B*. *fragilis* 638R, the TQO coded by *doxDA* is responsible for conversion of thiosulfate into tetrathionate, strengthening even more the relation between *bmoR* and *trxP* operons.

Previous studies were not able to establish a role for TrxP in *B*. *fragilis*^13,24^, but it has been suggested that the TQO coded by *doxDA* could be a potential substrate of TrxP due to its genomic location (it is located directly upstream of *trxP*) and organization of its cysteine residues^24^. Although *trxP* can be transcribed into a monocistronic mRNA^13^ and act independently, it is also a part of an operon with BF638R_2699 and *doxDA*, and our results show that these 3 genes are upregulated together in the *bmoR* mutant strain. BF638R_2699 encodes a hypothetical protein with no conserved domains, but the TQO coded by *doxDA* contain two conserved motifs, DoxD and DoxA. TQOs have been associated with the reduction of O_2_ from oxidation of thiosulfate in *Acidanus ambivalens*, with both of its subunits, DoxD and DoxA, previously described as part of the cytochrome c-oxidase complex^31^.

TrxP has been used previously as a model to show that successive cycles of oxidation and reduction of disulfide bonds can be used to achieve correct protein folding *in vivo*^24^. The formation of disulfide bonds is a critical step for proper function of many proteins in bacteria and correct disulfide bond formation, at least in *E*. *coli*, is obtained with the help of oxidation (DsbA/DsbB) and isomerization (DsbC/DsbD) pathways^32^. However, the aforementioned study showed that *B*. *fragilis* TrxP could act as both an oxidase as a reductase and that heterologously expressed TrxP was able to complement a *dsbC* mutant *E*. *coli*^24^. A protein with DsbC/DsbD domain is encoded in *B*. *fragilis* genome by BF638R_3040. No significant difference in BF638R_3040 expression could be observed in our transcriptomic analysis though, even after significant upregulation of *trxP* in Δ*bmoR* strain. Even though TrxP can complement DsbC in *E*. *coli*, a functional relation between these two may not exist in *B*. *fragilis*.

The thioredoxin system is considered the main system for thiol/disulfide redox balance in *B*. *fragilis*, since the species has an extensive repertoire of Trxs, but no described glutathione (GSH) or glutaredoxin^13,33^. However, the main portion of BF638R_0572 (~50%), codes for a CoA-disulfide reductase domain. CoA-disulfide reductases have been reported as responsible for keeping CoA in its reduced state (CoASH)^34^. CoASH is described as the sole low molecular weight thiol in some bacterial species and it is possible that CoASH could assume the role of GSH in the thiol/disulfide balance of *B*. *fragilis* as observed in other bacteria^34^. Disk diffusion assays showed that absence of BF638R_0572 increases sensitivity to diamide, a thiol oxidizing agent that was first described in the conversion of reduced glutathione to its disulfide conformation^35^. Therefore, an increase in sensitivity to this oxidant reflects an impairment in thiol/disulfide homeostasis inside the cell caused by deletion of BF638R_0572.

A previous work from our group showed that *bmoR* inactivation was associated with a decreased resistance to hydrogen peroxide^16^, but our current results shows that this increased susceptibility to H_2_O_2_ may not be directly related to alterations on peroxide detoxification pathway. Microarray analysis showed that deletion of *bmoR* had no significant impact in the expression of the major peroxide detoxifying enzymes, like catalase and alkyl hydroperoxide reductase, during exposure of *B*. *fragilis* to oxidative stress conditions. Phenotypic assays corroborated those results, showing that absence of neither *bmoR* nor BF638R_0572 seems to affect survival during prolonged oxygen exposure. Scavenging enzymes are crucial for detoxification of oxidants and, consequently, *B*. *fragilis* ability to survive for days in the presence of atmospheric oxygen^5^. Although O_2_ survival is not affected by *bmoR* mutation, our previous work showed that inactivation of *bmoR* impaired *B*. *fragilis* growth in oxidized media and soft agar tubes exposed to atmospheric oxygen, as well as growth recovery when challenged with hydrogen peroxide^16^. The fact that absence of *bmoR* affects growth, but not survival in oxidizing conditions strengthen the hypothesis that BmoR regulation in the cell during oxidative stress is more related to redox balance than detoxification of oxidants. The increased susceptibility to H_2_O_2_ described in our previous work reflects the burden of the oxidative stress in a strain deficient for enzymes important for redox balance.

Examination of *B*. *fragilis* regulatory mechanisms during oxidative stress conditions continues to expose the complexity of the species OSR. Here, we demonstrated that, unlike OxyR, BmoR role in *B*. *fragilis* OSR is more related to maintenance of a reducing state inside the cell than to regulation of scavenging enzymes. BmoR is responsible for controlling the expression of the pyridine nucleotide-disulfide oxidoreductase coded by BF638R_0572, which in turn seems to affect the expression of a thioredoxin operon. Though further work is needed to characterize BF638R_0572 or the *trxP* operon, understanding the regulatory network involved in the OSR of *B*. *fragilis* may be critical to determine how this anaerobic species is able to thrive during the infection process despite the oxidizing conditions found outside its normal intestinal environment.

## Methods

### Bacterial strains and growth conditions

Bacterial strains used in this study are listed in Table 4. *B*. *fragilis* strains were grown anaerobically on brain heart infusion broth (BHI) supplemented with hemin (5 µg/mL) at 37 °C. Rifampicin (20 µg/mL), gentamicin (100 µg/mL), tetracycline (5 µg/mL), cefoxitin (25 µg/mL) and erythromycin (10 µg/mL) were added to the media when necessary. *E*. *coli* strains were grown aerobically at 37 °C in LB broth. Ampicillin (100 µg/mL), tetracycline (10 µg/mL), kanamycin (50 µg/mL) and spectinomycin (50 µg/mL) were added when required.

**Table 4:** Bacterial strains used in this study.

Strains	Relevant phenotype/genotype^a^	Reference
***B***. ***fragilis***		
638R	Clinical isolate; Rif^r^ Gen^r^	^42^
FTB05	638R, Δ*bmoR::cfxA*, Cfx^r^ Rif^r^ Gen^r^	This Study
FTB08	638R, Δ*bmoR*Δ0572*::cfxA*, Cfx^r^ Tet^r^ Rif^r^ Gen^r^	This Study
FTB09	638R, Δ*bmoR::cfxA*, *bmoR*^+^, Cfx^r^ Rif^r^, Erm^r^ Gen^r^	This Study
***E***. ***coli***		
DH10B	Cloning host strain	Invitrogen
BL21	Cloning host strain	New England Biolabs Inc
BL21(DE3)	Expression host strain	New England Biolabs Inc
FTE06	BL21(DE3) carrying BmoR N-Terminus 6xHis-tag fusion peptide, Amp^r^	This Study

^a^Amp^r^, ampicillin resistance; Cfx^r^, cefoxitin resistance; Erm^r^, erythromycin resistance; Gen^r^, gentamicin resitance; Rif^r^, rifamycin resistanc.

### Construction of *B. fragilis* deletion mutant strains

#### Construction of Δ*bmoR*

Briefly, a 1.24 kb DNA fragment upstream of the *bmoR* gene containing approximately 30 bp of the N-terminal nucleotide sequence was amplified from *B*. *fragilis* 638 R chromosome by PCR using primers BmoR_BamHI_FOR and BmoR_BglII_REV (Table S1). The amplified product was cloned into the BamHI site of the suicide vector pFD516^36^ (Table S2) containing a cefoxitin gene cassette (*cfxA*) into the BamHI/EcoRI sites. A 1.17 kb DNA fragment downstream of *bmoR* containing approximately 120 bp internal C-terminal nucleotide sequence was amplified from *B*. *fragilis* 638 R chromosome by PCR using primers BmoR_EcoRI_FOR and BmoR_EcoRI_REV (Table S1). The amplified fragment was cloned into the EcoRI site of the pFD516cfxA construct containing the N-terminal fragment to make a Δ*bmoR*::*cfxA* deletion construct in pFD516. The resulting plasmid was mobilized from *E*. *coli* DH10B into *B*. *fragilis* 638 R by triparental mating^37^ and the transconjugants were selected on BHI containing 20 μg/ml of rifampicin, 100 μg/ml of gentamycin and 25 μg/ml of cefoxitin. Recombinants were tested for resistance to cefoxitin or erythromycin to select for double cross-over genetic recombination. Among recombinants resistant to cefoxitin and sensitive to erythromycin, the new strain FTB05 was selected for further studies.

#### Construction of Δ*bmoR* Δ0572 double mutant strain

A 1.52 kb DNA fragment upstream of BF638R_0572 gene containing approximately 110 nucleotides of the N-terminal internal sequence was amplified from *B*. *fragilis* 638R chromosome by PCR using primers BmOp_BamHI_FOR and BmOp_BglII_REV (Table S1). The amplified fragment was cloned into the BamHI site of pFD516 containing the *cfxA* gene into the BamHI/EcoRI sites as describe above. The 1.17 kb DNA fragment downstream of *bmoR* described above was cloned into the EcoRI site of the new plasmid to make a Δ*bmoR*Δ*0572::cfxA* double mutant construct in pFD516. The new plasmid was mobilized from *E*. *coli* DH10B into *B*. *fragilis* 638 R by triparental mating as describe above. Recombinants were tested for resistance to cefoxitin or erythromycin to select for double cross-over genetic recombination. Among recombinants resistant to cefoxitin and sensitive to erythromycin, the new strain FTB08 was selected for further studies.

#### Genetic complementation of Δ*bmoR* deletion mutant

A 418 bp DNA fragment containing *bmoR* gene ORF including 39 bp upstream of the ATG start codon was amplified from *B*. *fragilis* 638 R chromosome by PCR using primers BmoRBfExp_BamHI_FOR and BmoRBfExp_SstI_REV (Table S1). The promoterless *bmoR* gene was cloned into BamHI and SstI sites of the expression vector pFD340^38^ (Table S2). The new plasmid was mobilized from *E*. *coli* DH10B into *B*. *fragilis* FTB05 by triparental mating to obtain FTB09.

### Oxidative stress sensitivity assays

Survival during oxygen exposure was analyzed by measurement of the growth recovery after extended aerobic stress^25^. Cultures grown overnight were serially diluted in sterile phosphate-buffered saline (PBS) and 5 µL of each dilution was spotted onto multiple BHI agar plates. The plates were incubated at 37 °C under aerobic conditions and then transferred to anaerobic conditions after different exposure times (24, 48 and 72 h). The lowest dilution where colonies could be detected (more than five CFU) was used to compare the different survival capacity of each strain.

Disk diffusion assays to test for sensitivity to thiol oxidation by diamide was performed as previously described^39^. Briefly, 100 µL of overnight cultures were spread onto BHI plates and a sterile 6 mm filter disk containing 10 µL of 2 M diamide was placed onto the center of each plate. One duplicate set of plates was incubated at 37 °C for 48 h under anaerobiosis and the other duplicate set was exposed to air for 6 h followed by 42 h incubation in anaerobiosis, both at 37 °C. Diameter of the growth inhibition zone was measured after the incubation period and the results are the average of different experiments done in triplicate.

### RNA isolation and expression analysis

One hundred milliliters of mid-log phase (OD_550_ = 0.3) cultures of wild-type and mutant strains were used for total RNA extraction using the hot phenol method as previously described^40^. Part of the cultures were exposed to atmospheric oxygen for 1 h under agitation (250 rpm) at 37 °C prior to the RNA extraction to induce an OSR. Total bacterial RNA was extracted by the hot-phenol method and total RNA was purified using the RNeasy Mini Kit (QIAGEN). Purified RNA was treated with Turbo DNase (2 U/µl) to remove residual DNA according to manufacturer’s instruction (Invitrogen Inc.). cDNA synthesis was performed by reverse transcription reaction using SuperScript III Reverse Transcriptase (Invitrogen). The cDNA library created was treated with an RNase cocktail (500 U/mL of RNase A; 20,000 U/mL of RNase T1; Invitrogen Inc.) to degrade template RNA according to Affymetrix GeneChip expression technical manual. Total cDNA was purified by phenol/chloroform extraction and partially fragmented with DNAse I to obtain fragmented cDNA in the 50 to 200 bp range. The fragmented cDNA was labelled with terminal bitoin-ddUTP (Enzo Life Sciences) using terminal deoxynucleotidyl transferase according to Affymetrix GeneChip expression technical manual. Microarray analysis was performed using Affymetrix GeneChip array (format 81/4). Labelled cDNA was hybridized and processed by the University of North Carolina at Chapel Hill Functional Genomics Core. Microarray data were submitted to NCBI’s Gene Expression Omnibus (Edgar *et al*., 2002) and are accessible through GEO accession number GSE104575 (https://www.ncbi.nlm.nih.gov/geo/query/acc.cgi?acc=GSE104575).

Quantitative real-time RT-PCR was performed as previously described^39^. Primers were designed to amplify 100–150 bp fragments of each gene (Table S1) in a 15 µL standard reaction mixture with 7.5 µL of iQ SYBR Green Supermix (2x; Bio-Rad), 1.5 µL of each primer (5 mM) and 25 ng of cDNA. Amplification was carried out using an iCycler Real-Time Detection System (Bio-Rad) with all sample reactions running in triplicate. Data obtained were normalized using 16 S rRNA expression levels as reference and relative expression was determined by calculating the ΔΔCq for each gene. Results are displayed as fold induction relative to wild-type strain grown in anaerobiosis.

### Protein expression and purification

A 435 bp DNA fragment containing the entire *bmoR* gene ORF was amplified from *B*. *fragilis* 638 R chromosome by PCR using primers BmoRexp_NdeI_FOR and BmoRexp_BamHI_REV containing NdeI and BamHI restriction sites respectively (Table S2). The amplified 435 bp DNA fragment was cloned in-frame into the NdeI and BamHI sites of the expression vector pET16b (Novagen) to produce a recombinant BmoR protein containing an N-terminus 6xHis fused peptide tag. The new construct was purified from *E*. *coli* BL21 (New England BioLabs Inc) and electroporated into *E*. *coli* BL21 (DE3) (New England BioLabs Inc) to obtain *E*. *coli* FTE06 (Table 4). For recombinant protein expression, *E*. *coli* FTE06 was grown in LB broth containing 100 µg/mL ampicillin at 37 °C in a rotatory shaker to an OD_550nm_ of approximately 0.4. Then, 0.5 mM IPTG was added into the culture media for induction of the recombinant protein expression and incubated at 30 °C for 4 h before bacterial harvesting by centrifugation. Bacterial pellet was suspended in lysis buffer (50 mM NaH_2_PO_4_, 500 mM NaCl and 5 mM imidazole; pH 8,0) and RNase A (5 µg/mL) and DNase I (1 µg/mL) were added to the suspension. Bacterial cell lysis were carried out on a French press cell disruptor and cell debris was removed by centrifugation. The cell-free clear supernatant was then mixed with Co-NTA agarose (Cube Biotech) beads equilibrated in lysis buffer and incubated on a platform rocker for 2 hours under low rotation (150 rpm) for binding of His-tagged BmoR to Co-NTA agarose beads. After incubation, the suspension was transferred to a disposable chromatography column. The column was washed with lysis buffer containing 5 mM immidazole. The column was washed with lysis buffer with increasing stepwise concentrations of imidazole (10 mM, 20 mM and 40 mM). Recombinant His-tagged BmoR was eluted from the column with 200 mM imidazole in lysis buffer. The washing and elution fractions were analyzed by SDS-PAGE to estimate protein purification quality. The purified recombinant BmoR, with a molecular weight of approximately 15.47 kDa, was concentrated using Amicon 8050 Stirred Ultrafiltration Cell (Millipore) with an YM 10 MW membrane. The concentrate was dialyzed in 8,000 MWCO BioDesignDialysis Tubing (BioDesing, Inc) at 4 °C for 3 days against 0.1x PBS. Proteins concentration was determined by absorbance at 280 nm in a spectrophotometer (ND-1000, NanoDrop).

### Electrophoretic mobility shift assay (EMSA)

Eletrophoretic mobility shift assay performed as previously described^41^, with some modifications was used to determine protein-DNA interactions of recombinant 6xHis-tagged BmoR to target dsDNA fragments containing promoter regions for *bmoR* (151 bp), hypothetical protein BF638R_2699 (171 bp) or lipoprotein BF638R_4199 (161 bp), respectively. The target DNAs were amplified from *B*. *fragilis* 638 R chromosome by PCR using the following primers (Table S1): BmoR_Shift01_FOR and BmoR_Shift01_REV (*bmoR*); 2699_Shift_FOR and 2699_Shift_REV (BF638R_2699); and 4194_Shift_FOR and 4194_Shift_REV (BF638R_4199). A non-competitor dsDNA fragment of 101 bp was amplified by PCR from *hlyD* gene using primers HlyD-forward and HlyD-reverse (Table S1). Briefly, a 20 µL reaction mixture was set up with 15 nM of dsDNA, 400 nM of purified BmoR protein (0 to 500 nM) in binding buffer (60 mMKCl, 0.5 mM EDTA, 1 mM DTT, 12% glycerol, 0.1 gm/mL BSA and 20 mM HEPES, pH 7.9) and was incubated at room temperature for 20 min. Samples were then loaded onto an 8% nondenaturing polyacrylamide gel and were run in 1x TBE buffer (89 mM Tris, 89 mM boric acid, 50 mM EDTA, pH 8.0) at 100 V for 1 h. Gels were stained with SYBR gold for 20 min and DNA bands were visualized under Ultra-Violet light. The concentration of purified recombinant BmoR (400 nM) used was determined from a stepwise concentration assay (0–500 nM) to estimate optimal BmoR binding ratio to its target DNA by EMSA analysis.

## Electronic supplementary material

Supporting information

Table S3

Table S4

Table S5

Table S6

## References

[1] Giamarellou H (2000). Anaerobic infection therapy. Int. J. Antimicrob. Agents.

[2] Baughn AD, Malamy MH (2004). The strict anaerobe Bacteroides fragilis grows in and benefits from nanomolar concentrations of oxygen. Nature.

[3] Sund CJ (2008). The Bacteroides fragilis transcriptome response to oxygen and H2O2: the role of OxyR and its effect on survival and virulence. Mol. Microbiol..

[4] Mishra S, Imlay JA (2013). An anaerobic bacterium, Bacteroides thetaiotaomicron, uses a consortium of enzymes to scavenge hydrogen peroxide. Mol. Microbiol..

[5] Rocha ER, Selby T, Coleman JP, Smith CJ (1996). Oxidative stress response in an anaerobe, Bacteroides fragilis: a role for catalase in protection against hydrogen peroxide. J. Bacteriol..

[6] Rolfe RD, Hentges DJ, Campbell BJ, Barrett JT (1978). Factors related to the oxygen tolerance of anaerobic bacteria. Appl. Environ. Microbiol..

[7] Rolfe RD, Hentges DJ, Barrett JT, Campbell BJ (1977). Oxygen tolerance of human intestinal anaerobes. Am. J. Clin. Nutr..

[8] Herren CD, Rocha ER, Smith CJ (2003). Genetic analysis of an important oxidative stress locus in the anaerobe Bacteroides fragilis. Gene.

[9] Rocha ER, Smith CJ (1999). Role of the alkyl hydroperoxide reductase (ahpCF) gene in oxidative stress defense of the obligate Anaerobe bacteroides fragilis. J. Bacteriol..

[10] Carlsson J, Wrethén J, Beckman G (1977). Superoxide dismutase in Bacteroides fragilis and related Bacteroides species. J. Clin. Microbiol..

[11] Betteken MI, Rocha ER, Smith CJ (2015). Dps and DpsL Mediate Survival *In Vitro* and *In Vivo* during the Prolonged Oxidative Stress Response in Bacteroides fragilis. J. Bacteriol..

[12] Rocha ER, Smith CJ (2004). Transcriptional regulation of the Bacteroides fragilis ferritin gene (ftnA) by redox stress. Microbiol. Read. Engl..

[13] Reott MA, Parker AC, Rocha ER, Smith CJ (2009). Thioredoxins in redox maintenance and survival during oxidative stress of Bacteroides fragilis. J. Bacteriol..

[14] Rocha ER, Herren CD, Smalley DJ, Smith CJ (2003). The complex oxidative stress response of Bacteroides fragilis: the role of OxyR in control of gene expression. Anaerobe.

[15] Rocha ER, Owens G, Smith CJ (2000). The redox-sensitive transcriptional activator OxyR regulates the peroxide response regulon in the obligate anaerobe Bacteroides fragilis. J. Bacteriol..

[16] Teixeira FL (2013). The role of BmoR, a MarR Family Regulator, in the survival of Bacteroides fragilis during oxidative stress. Int. J. Med. Microbiol. IJMM.

[17] George AM, Levy SB (1983). Gene in the major cotransduction gap of the *Escherichia coli* K-12 linkage map required for the expression of chromosomal resistance to tetracycline and other antibiotics. J. Bacteriol..

[18] George AM, Levy SB (1983). Amplifiable resistance to tetracycline, chloramphenicol, and other antibiotics in *Escherichia coli*: involvement of a non-plasmid-determined efflux of tetracycline. J. Bacteriol..

[19] Perera IC, Grove A (2010). Molecular mechanisms of ligand-mediated attenuation of DNA binding by MarR family transcriptional regulators. J. Mol. Cell Biol..

[20] Sulavik MC, Gambino LF, Miller PF (1995). The MarR repressor of the multiple antibiotic resistance (mar) operon in *Escherichia coli*: prototypic member of a family of bacterial regulatory proteins involved in sensing phenolic compounds. Mol. Med. Camb. Mass.

[21] Wilkinson SP, Grove A (2004). HucR, a novel uric acid-responsive member of the MarR family of transcriptional regulators from Deinococcus radiodurans. J. Biol. Chem..

[22] Teixeira, F. L., Domingues, R. M. C. P. & Lobo, L. A. Regulation of oxidative stress–related genes implicated in the establishment of opportunistic infections by Bacteroides fragilis. In *Stress and Environmental Regulation of Gene Expression and Adaptation in Bacteria* 1416 (Wiley-Blackwell, 2016).

[23] Silva CMG (2018). Inactivation of MarR gene homologs increases susceptibility to antimicrobials in Bacteroides fragilis. Braz. J. Microbiol. Publ. Braz. Soc. Microbiol..

[24] Shouldice SR (2010). *In vivo* oxidative protein folding can be facilitated by oxidation-reduction cycling. Mol. Microbiol..

[25] Ndamukong IC, Gee J, Smith CJ (2013). The extracytoplasmic function sigma factor EcfO protects Bacteroides fragilis against oxidative stress. J. Bacteriol..

[26] Cohen SP, Hächler H, Levy SB (1993). Genetic and functional analysis of the multiple antibiotic resistance (mar) locus in *Escherichia coli*. J. Bacteriol..

[27] Martin RG, Rosner JL (1995). Binding of purified multiple antibiotic-resistance repressor protein (MarR) to mar operator sequences. Proc. Natl. Acad. Sci. USA.

[28] Chng S-S (2012). Overexpression of the rhodanese PspE, a single cysteine-containing protein, restores disulphide bond formation to an *Escherichia coli* strain lacking DsbA. Mol. Microbiol..

[29] Nandi DL, Horowitz PM, Westley J (2000). Rhodanese as a thioredoxin oxidase. Int. J. Biochem. Cell Biol..

[30] Liu L-J (2014). Thiosulfate transfer mediated by DsrE/TusA homologs from acidothermophilic sulfur-oxidizing archaeon Metallosphaera cuprina. J. Biol. Chem..

[31] Müller FH (2004). Coupling of the pathway of sulphur oxidation to dioxygen reduction: characterization of a novel membrane-bound thiosulphate:quinone oxidoreductase. Mol. Microbiol..

[32] Bader MW (2001). Turning a disulfide isomerase into an oxidase: DsbC mutants that imitate DsbA. EMBO J..

[33] Rocha ER, Tzianabos AO, Smith CJ (2007). Thioredoxin reductase is essential for thiol/disulfide redox control and oxidative stress survival of the anaerobe Bacteroides fragilis. J. Bacteriol..

[34] Mallett TC (2006). Structure of coenzyme A-disulfide reductase from Staphylococcus aureus at 1.54 A resolution. Biochemistry (Mosc.).

[35] Kosower NS, Kosower EM, Wertheim B, Correa WS (1969). Diamide, a new reagent for the intracellular oxidation of glutathione to the disulfide. Biochem. Biophys. Res. Commun..

[36] Smith CJ, Rollins LA, Parker AC (1995). Nucleotide sequence determination and genetic analysis of the Bacteroides plasmid, pBI143. Plasmid.

[37] Shoemaker NB, Getty C, Gardner JF, Salyers AA (1986). Tn4351 transposes in Bacteroides spp. and mediates the integration of plasmid R751 into the Bacteroides chromosome. J. Bacteriol..

[38] Smith CJ, Rogers MB, McKee ML (1992). Heterologous gene expression in Bacteroides fragilis. Plasmid.

[39] Sund CJ, Wells WG, Smith JC (2006). The Bacteroides fragilis P20 scavengase homolog is important in the oxidative stress response but is not controlled by OxyR. FEMS Microbiol. Lett..

[40] Aiba H, Adhya S, de Crombrugghe B (1981). Evidence for two functional gal promoters in intact *Escherichia coli* cells. J. Biol. Chem..

[41] Hao Z (2014). The multiple antibiotic resistance regulator MarR is a copper sensor in *Escherichia coli*. Nat. Chem. Biol..

[42] Privitera G, Dublanchet A, Sebald M (1979). Transfer of multiple antibiotic resistance between subspecies of Bacteroides fragilis. J. Infect. Dis..

