# Supplementary material for: Deletion of BmoR affects the expression of genes related to thiol/disulfide balance in Bacteroides fragilis
Source: Sci Rep. 2018 Sep 26;8:14405. doi: 10.1038/s41598-018-32880-7 (PMC6158253; doi:10.1038/s41598-018-32880-7)
Supplement: Supplementary file 1 — Supporting information [file 41598_2018_32880_MOESM1_ESM.docx]

***Supplementary Information***

**Deletion of BmoR affects the expression of genes related to thiol/disulfide balance in *Bacteroides fragilis***

Felipe L. Teixeira ^1,2,*^, Heidi Pauer ^1,2^, Scarlathe B. Costa ^1^, C. Jeffrey Smith ^2^, Regina M.C.P Domingues ^1^, Edson R. Rocha ^2,+^, Leandro A. Lobo^1,*,+^

^1^ Departamento de Microbiologia Médica, Instituto de Microbiologia Paulo de Góes, Universidade Federal do Rio de Janeiro, Rio de Janeiro RJ, Brasil

^2^ Department of Microbiology and Immunology, Brody School of Medicine, East Carolina University, Greenville NC, USA

^*^ Corresponding authors: Felipe L. Teixeira (teixeirafl@micro.ufrj.br) or Leandro A. Lobo (lobol@micro.ufrj.br) / Tel.: +55 21 2560-8344 / Fax.: +55 21 2560-8028

^+^ These authors contributed equally to this work


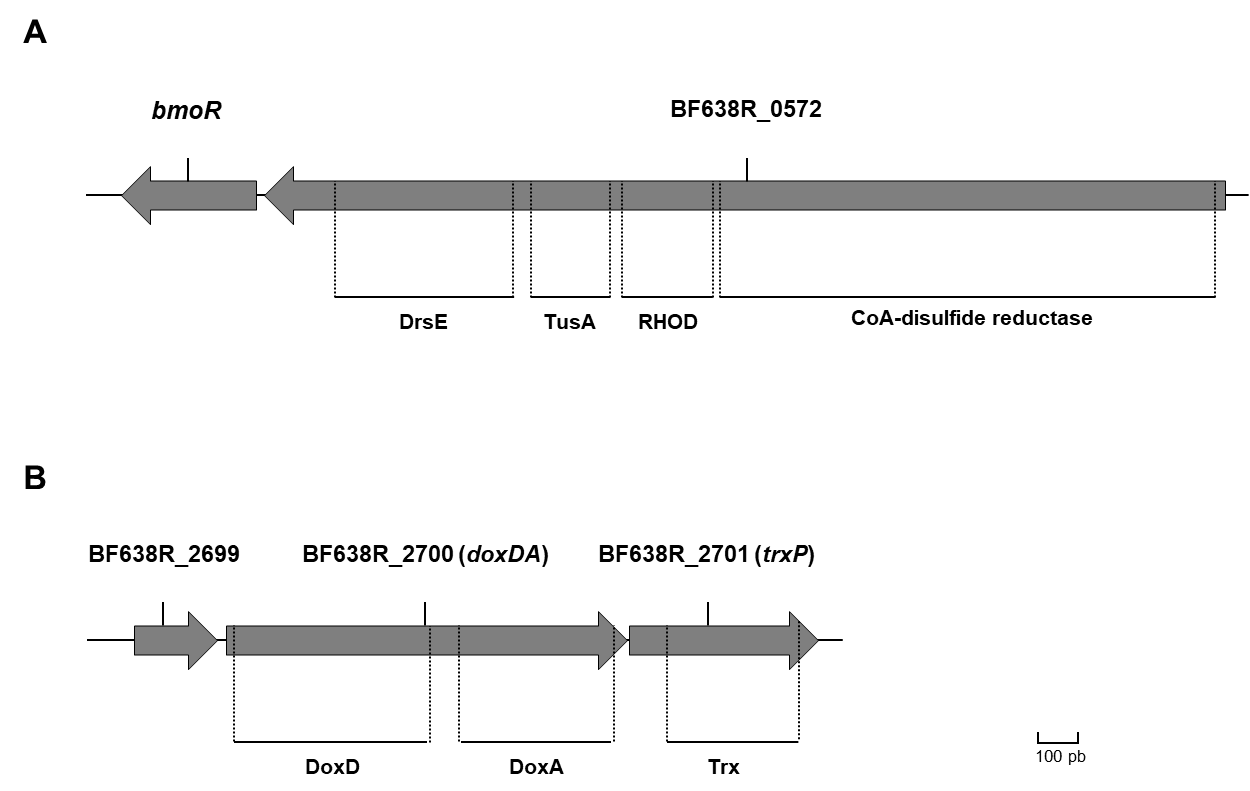


**Figure S1:** Genetic organization of (A) *bmoR* and (B) *trxP* operons. Genes are shown as gray arrows. Connected dashed lines represent domain ranges relative to the protein encoded by each gene, as seen on table 3. CoA: coenzyme A; DoxA: TQO small subunit DoxA; DoxD: TQO small subunit DoxD; DrsR: DsrE/DsrF/DrsH-like family; RHOD: rhodanese-like; TusA: Sulfurtransferase TusA; Trx: thioredoxin


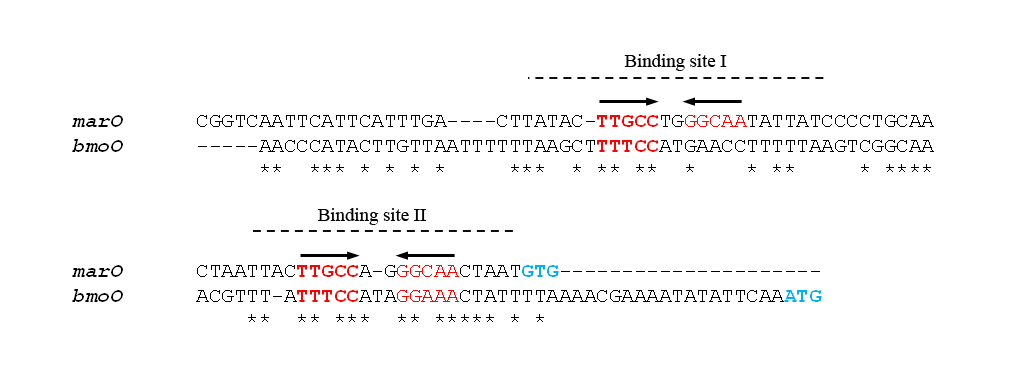


**Figure S2:** Sequence alignment between *E. coli* MarR operator (*marO*) and part of the promoter region of BF638R_0572 (*bmoO*) used as DNA probe on EMSA. The arrows show the inverted repeats, with conserved pentanucleotides marked in red. Dashed lines represent MarR binding sites in *E. coli.* The * indicates an identical base pair in the alignment. Nucleotides marked in blue represent the start codon for each gene.

**Table S1:** Primers used in this study.

| **Primer** | **Sequence** | **Amplicon (bp)** | **Restriction sites added**^1^ |
| --- | --- | --- | --- |
| **Deletion** |  |  |  |
| BmoR_BamHI_FOR  BmoR_BglII_REV | 5' CAGCAAAGGATCCCGTAGCC 3'  5' GCTTTGTAGATCTCGCGCATC 3' | 1,237 | BamHI  BglII |
| BmoR_EcoRI_FOR  BmoR_EcoRI_REV | 5' CCGACGGGAATTCGGTGAAGTAG 3'  5' CAGCCTGGAATTCTGCCAGG 3' | 1,175 | EcoRI |
| BmOp_BamHI_FOR  BmOp_BglII_REV | 5' CGTTTCCGGATCCTATGGAAC 3'  5' TGGCATAAGATCTGTACTTTCC 3' | 1,525 | BamHI  BglII |
| **BmoR complementation** |  |  |  |
| BmoRBfExp_BamHI_FOR  BmoRBfExp_SstI_REV | 5' GATAATGCTAACATGGATCCGTTTATT 3'  5'ACCTGTGGTGAGCTCTGATGTT 3' | 418 | BamHI  SstI |
| **Recombinant BmoR** |  |  |  |
| BmoRexp_NdeI_FOR  BmoRexp_BamHI_REV | 5' AATAAGGACATATGAATACAATATG 3'  5' CAAATGGATCCCTGTCTGTATGG 3' | 435 | NdeI  BamHI |
| **EMSA** |  |  |  |
| *Target DNAs:* |  |  |  |
| BmoR_Shift01_FOR  BmoR_Shift01_REV | 5' GCACTTCCGTAAGTAAGATAACC 3'  5'CCTGCTACGCCTCCAATAATAAT 3' | 151 | - |
| 2699_Shift_FOR  2699_Shift_REV | 5' TGTTTTCATGCGTCACATAGCC 3'  5' ACGAGACCGATAAATGTGAGTC 3' | 171 | - |
| 4194_Shift_FOR  4194_Shift_REV | 5' CCGGCCTCCATATCAAACAATA 3'  5' ACACGGAACAAACTCCTGCAAA 3' | 161 | - |
| Non-*Competitor DNA*^2^*:* |  |  |  |
| HlyD-forward  HlyD-reverse | 5' ACTCCTCCTTCATGTCGTTTCAC 3'  5' CGTCTGACAGCAAGATAAAGTCC 3' | 101 | - |
| **RT-qPCR** |  |  |  |
| qPCR_0572_FOR  qPCR_0572_REV | 5' GCATATGTCAACAATCGGCCTC 3'  5' ATAGATACTTGCGCTCCCAGTG 3' | 107 | - |
| qPCR_TrxC_FOR  qPCR_TrxC_REV | 5' CACAATCGGGCACCATTCAC 3'  5' ATAGCCGGTTTGTCACCCAG 3' | 106 | - |
| qPCR_2699_FOR  qPCR_2699_REV | 5' GGTCTCGTCATTGGAGCTGTAG 3'  5' CCAAAGGGTACTGTTGACCGG 3' | 105 | - |
| qPCR_2700_FOR  qPCR_2700_REV | 5' AGTATCGGCGTATTCAGTCTGG 3'  5' GGCTACACCCAGAATACCGATC 3' | 102 | - |

^1^ Restriction sites added are underlined on primer sequences.

^2^ From Lobo *et al*., 2013.

**Table S2:** Plasmids used in this study.

| **Plasmid** | **Description^1^** | **Reference** |
| --- | --- | --- |
| pET16b | *E. coli* expression vector; Amp^r^ | Novagen |
| pFD340 | *B. fragilis* expression vector; Amp^r^, Erm^r^ | Smith, Rogers and McKee, 1992 |
| pFD516 | *B. fragilis* suicide vector; (Spec^r^), Erm^r^ | Smith, Rollins and Parker, 1995 |
| pFT08 | pFD516 carryin *ΔbmoR::cfxA* deletion construct. (Spect^r^), Cfx^r^, Erm^r^ | This study |
| pFT09 | pFD516 carryin *ΔbmoRΔ0572::cfxA* double mutant construct. (Spect^r^), Cfx^r^, Erm^r^ | This study |
| pFT10 | pFD340 carrying promoterless *bmoR* gene. Expression driven by the constitutive IS4351 promoter. (Amp^r^), Erm^r^ | This study |
| pFT11 | pET16b carrying promoterless *bmoR* gene fused to an N-terminus 6xHis peptide tag. Amp^r^ | This study |

^1^ Amp^r^, ampicillin resistance; Cfx^r^, cefoxitin resistance; Erm^r^, erythromycin resistance; Spec^r^, spectinomycin resistance; Tet^r^, tetracycline resistance. For *B. fragilis* suicide vectors, parentheses indicate antibiotic resistance expression in *E. coli*.

**Table S7:** Genes up- or down regulated in *B. fragilis bmoR* mutant strain in both anaerobiosis and after 1 hour of exposure to atmospheric oxygen

| **Gene ID** | **GenBank definition** | **Fold-change** | |
| --- | --- | --- | --- |
|  |  | **Anaerobic** | **O_2_ exposed** |
| *Upregulated* |  |  |  |
| BF638R_0572 | putative pyridine nucleotide oxidoreductase | 11.36 | 7.62 |
| BF638R_1629 | conserved hypothetical protein | 1.68 | 1.88 |
| BF638R_2700 | conserved hypothetical membrane protein | 1.98 | 7.41 |
| BF638R_4194 | putative lipoprotein | 1.27 | 6.69 |
|  |  |  |  |
| *Downregulated* |  |  |  |
| BF638R_0283 | putative exported alpha-galactosidase | 1.26 | 1.52 |
| BF638R_0786 | putative LPS biosynthesis related glycosyl transferase | 1.51 | 2.47 |
| BF638R_0919 | putative AraC transcriptional regulatory protein | 1.28 | 1.60 |
| BF638R_1035 | putative outer membrane protein | 1.27 | 1.39 |
| BF638R_2591 | putative polysaccharide transporter/flippase | 1.61 | 2.04 |
| BF638R_2598 | putative LPS biosynthesis related DNTP-hexose dehydratase-epimerase | 1.93 | 1.74 |
| BF638R_3476 | putative LPS biosynthesis related glycosyltransferase | 1.89 | 1.64 |
| BF638R_3485 | putative LPS biosynthesis-related sugar-phosphate nucleotidyltransferase | 1.97 | 1.06 |
| BF638R_4283 | hypothetical protein | 1.46 | 1.17 |

**References**

Lobo, L.A., Jenkins, A.L., Jeffrey Smith, C. and Rocha, E.R. (2013). Expression of *Bacteroides fragilis* hemolysins *in vivo* and role of HlyBA in an intra-abdominal infection model. *Microbiology open* **2**: 326-337.

Smith, C.J., Rogers M.B., and McKee, M.L. (1992). Heterologous gene expression in *Bacteroides fragilis*. *Plasmid* **27**: 141-154.

Smith, C.J., Rollins, L.A., and Parker, A.C. (1995). Nucleotide sequence determination and genetic analysis of the *Bacteroides* plasmid, pBI143. *Plasmid* **34**: 211-222.
